# Learning-Related Brain-Electrical Activity Dynamics Associated with the Subsequent Impact of Learnt Action-Outcome Associations

**DOI:** 10.3389/fnhum.2017.00252

**Published:** 2017-05-15

**Authors:** Fabian Baum, Uta Wolfensteller, Hannes Ruge

**Affiliations:** Department of Psychology, Technische Universität DresdenDresden, Germany

**Keywords:** intention, prediction, ideomotor learning, instrumental learning, perceptual learning, sensory attenuation, multi-sensory integration

## Abstract

Goal-directed behavior relies on the integration of anticipated outcomes into action planning based on acquired knowledge about the current contingencies between behavioral responses (R) and desired outcomes (O) under specific stimulus conditions (S). According to ideomotor theory, bidirectional R-O associations are an integral part of this knowledge structure. Previous EEG studies have identified neural activity markers linked to the involvement of such associations, but the initial acquisition process has not yet been characterized. The present study thus examined brain-electrical activity dynamics during the rapid acquisition of novel bidirectional R-O associations during instructed S-R learning. Within a trial, we inspected response-locked and stimulus-locked activity dynamics in order to identify markers linked to the forward and backward activation of bidirectional R-O associations as they were being increasingly strengthened under forced choice conditions. We found that a post-response anterior negativity following auditory outcomes was increasingly attenuated as a function of the acquired association strength. This suggests that previously reported action-induced sensory attenuation effects under extensively trained free choice conditions can be established within few repetitions of specific R-O pairings under forced choice conditions. Furthermore, we observed the even more rapid development of a post-response but pre-outcome fronto-central positivity which was reduced for high R-O learners which might indicate the rapid deployment of preparatory attention towards predictable outcomes. Finally, we identified a learning-related stimulus-locked activity modulation within the visual P1-N1 latency range which might reflect the multi-sensory integration of the perceived antecedent visual stimulus the anticipated auditory outcome.

## Introduction

Behavior is considered goal-directed when an actor integrates information about the anticipated outcome into ongoing action planning (Dickinson and Balleine, 1994). Outcome integration requires the prior acquisition of knowledge about the current contingencies between behavioral responses (R) and their outcomes (O) under specific antecedent stimulus conditions (S). Specifically, the successful acquisition of novel S-R-O contingency representations enables action selection through an S-O, O-R association chain as one possible route to goal-directed action (Trapold, 1970; Urcuioli, 2005; Balleine and Ostlund, 2007; de Wit and Dickinson, 2009). According to ideomotor theory, this is possible as the contingency between an R and the ensuing O is encoded as a bi-directional association where “forward” R-O associations and “backward R-O” (i.e., O-R) associations are regarded as two sides of the same coin (Greenwald, 1970b; Urcuioli, 2005; de Wit and Dickinson, 2009; Shin and Proctor, 2012; Waszak et al., 2012). Hence, a specific outcome representation can be activated by planning a specific action (via forward R-O) whereas a specific action representation can be activated by perceiving or anticipating a specific outcome (via backward R-O).

The present study aimed at characterizing learning-related neural activity changes *across trials* associated with the initial formation of bi-directional R-O associations in a forced choice context where actions are triggered by an antecedent stimulus. Moreover, by exploiting the excellent temporal resolution of the EEG signal, we could assess learning-related activity changes across different phases *within a trial*. On the one hand, this included learning-related modulations of post-response event-related potentials (ERPs) associated with the *forward* activation of bidirectional R-O associations and the ensuing altered perception of predicted outcomes. On the other hand, this included learning-related modulations of pre-response ERPs associated with the *backward* activation of bidirectional R-O associations via the stimulus-triggered anticipation of future outcomes.

In different ways, previous ERP studies have identified neural markers reflecting the involvement of bidirectional R-O associations, but to our knowledge, none of these studies has examined forward or backward R-O activation processes as they are developing across the initial learning trials. Instead, previous studies examined ERP markers reflecting how already well learned R-O associations impacted response selection or post-response outcome processing. Related to this, previous behavioral and ERP studies have typically employed experimental settings in which R-O associations were acquired and/or probed under free choice conditions. Presumable, this design choice often followed the reasoning that free choice conditions would encourage subjects to adopt an intention-based action mode involving R-O associations whereas forced-choice conditions would induce a stimulus-based or habitual action mode predominantly relying on S-R associations (Herwig et al., 2007; Krieghoff et al., 2011; Pfister et al., 2011). However, other recent studies suggest that forced choice conditions are detrimental for R-O integration only if extensive S-R-O practice allows for a considerable level of habitualization, that is, reliance on S-R associations. These studies have shown that R-O associations are learned and do impact subsequent behavior in entirely forced-choice experimental settings with acquisition phases involving less than 10 repetitions of specific S-R-O combinations (Wolfensteller and Ruge, 2011, 2014; Ruge et al., 2012; Ruge and Wolfensteller, 2013). Hence, under these circumstances, subjects seem to stick to an intention-based action mode as substantial habitualization could not develop within this limited amount of practice (Wolfensteller and Ruge, 2012). Accordingly, we hypothesized that we should be able to observe—under forced choice conditions—within the first few S-R-O learning trials the emergence of R-O-related ERP markers similar to those that have previously been identified after extended R-O learning periods under free choice conditions.

The most extensively studied ERP marker of R-O-related processes was identified in a class of studies that examined the so-called action-induced sensory attenuation effects as a manifestation of *post-response* ERP modulations attributed to the forward activation of bidirectional R-O associations (Waszak et al., 2012). The action-induced sensory attenuation effect denotes the altered perception of stimuli which are predictably triggered by one’s own actions (i.e., action outcomes). Specifically, action outcomes are perceived to be attenuated and shifted in time compared to stimuli that are unpredicted or predicted by other stimuli rather than one’s own actions (Von Holst and Mittelstaedt, 1950; Weiskrantz et al., 1971; Blakemore et al., 1998; Haggard et al., 2002). This is a widespread phenomenon that has not only been assessed behaviorally but also neuro-physiologically using different techniques (Schafer and Marcus, 1973; McCarthy and Donchin, 1976; Martikainen et al., 2005; Aliu et al., 2009; Reznik et al., 2014; Timm et al., 2014), including a number of recent EEG studies (Lange, 2011; Desantis et al., 2012; Hughes et al., 2013a,b; SanMiguel et al., 2013; Mifsud et al., 2016; Timm et al., 2016). When outcomes are sounds as in most previous studies, the typical finding are reduced outcome-evoked ERP amplitudes within the latency range of the N1 and P2 components at fronto-central electrodes.

Another class of studies examined the backward activation of bi-directional R-O associations prior to responding instead of the action-induced forward activation of the same associations. One prominent paradigm that has been used to explore this type of processes in behavioral studies is the outcome-induced response priming paradigm in which the *perception* of previously learnt action outcomes biases current response selection towards the action that produced that outcome in a preceding learning phase (Greenwald, 1970a; Hommel, 1996; Elsner and Hommel, 2001). Another behavioral paradigm identified similar response selection biases due to the pre-response *anticipation* of outcomes and the ensuing backward activation of bidirectional R-O associations (Kunde, 2001; Pfister et al., 2010). Moreover, it has been suggested that free choice conditions as compared to stimulus-based forced choice conditions are associated with a stronger anticipatory backward activation of bidirectional R-O associations (Krieghoff et al., 2011). ERP studies suggested that this is reflected by a more pronounced readiness-potential-like component as a general marker of more intention-based action planning (Waszak et al., 2005; Krieghoff et al., 2011). We hypothesized that we might observe a similar ERP amplification even under forced choice conditions as stimulus-based response selection would still be intentional in nature due to weak habitualization within the limited number of only eight S-R-O repetitions (Wolfensteller and Ruge, 2012; Ruge and Wolfensteller, 2013).

We employed an experimental design derived from the differential outcome paradigm (Trapold, 1970; Colwill and Rescorla, 1985; Elsner and Hommel, 2001; Noonan et al., 2011) where differential auditory response outcomes were presented during instruction-based visuo-motor learning (Wolfensteller and Ruge, 2014; Ruge and Wolfensteller, 2015). Specifically, each distinct link between a visual stimulus and a manual response was predictably followed by a distinct auditory outcome. To obtain sufficient data for initial learning trials, each subject worked through 10 different blocked learning episodes each comprising a novel and unique set of visual stimuli and auditory outcomes. Different from related previous studies, to minimize cross-talk between learning blocks, we used a total of 40 distinct sound outcomes that were easily discriminable natural sounds as compared to the typically employed simple sine-wave tones. Each learning episode comprised eight learning trials for each of the four unique S-R-O triples. From previous behavioral studies, it is known that eight repetitions of specific R-O pairings are sufficient to establish durable bidirectional R-O associations as assessed by post-learning O-R compatibility effects (Wolfensteller and Ruge, 2011, 2014; Ruge et al., 2012). This behavioral index exploits outcome-triggered response priming effects which can be observed when a previously learnt action outcome subsequently becomes an imperative stimulus that requires either the response which produced that outcome in the preceding learning phase (O-R compatible) or a response which produced a different outcome (O-R incompatible). Such O-R compatibility effects—expressed in prolonged response times and increased error rates in incompatible trials compared to compatible trials—are commonly observed and indicate that the perception of an outcome automatically primes the action it was previously produced by Greenwald (1970a), Hommel (1996) and Elsner and Hommel (2001). We used this post-learning O-R compatibility effect as a proxy for bidirectional R-O association strength acquired during the preceding S-R-O learning phase. Our rationale was to use this behavioral index in a correlation analysis to identify correlated learning-related ERP modulations that are specifically related to the increasing involvement of bidirectional R-O associations across the preceding S-R-O learning phase.

To assess how quickly ERPs would start being modulated by the integration of bidirectional R-O associations, we analyzed learning-related ERP activation dynamics on two timescales. First, very rapid learning was assessed from the first to the second repetition of specific R-O links within learning blocks. This comparison involved first repetition trials (defined as the first occurrence of specific R-O links) which were special in the sense that specific R-O links were entirely unknown until the outcome sounds were played the first time following correct response execution. Starting from second repetition trials, R-O links could theoretically be known in advance. Second, learning on the slower (but still relatively rapid) timescale was assessed from the second to the final eighth repetition of specific R-O links within learning blocks. This slower timescale comparison hence involved R-O repetitions that were all qualitatively quite similar and R-O-related learning processes were expected to change gradually across repetitions.

To summarize, based on the previous ERP literature sketched above, we hypothesized R-O-learning-related modulations of two response-locked ERP components, including an increasing post-response attenuation of sound-evoked ERPs and an increasing pre-response amplification of a readiness potential-like component. Additionally we were interested in modulations of stimulus-locked ERPs due to the special nature of the present study where action selection depended on a specific antecedent stimulus S in contrast to the typical free choice procedure employed in previous studies. Hence, the anticipatory backward activation of bidirectional R-O associations was assumed to be triggered by the antecedent stimulus via an S-O, O-R association chain which should be most clearly reflected in learning-related modulations of stimulus-locked rather than response-locked ERPs.

## Materials and Methods

### Subjects

Thirty-five subjects participated in this study. Three subjects were excluded due to insufficient raw data quality and two additional subjects were excluded due to excessive error rates greater than 20% in the unguided implementation trials (SRO repetitions 4–8). The mean age of the resulting 30 subjects was 23.0 years, ranging from 18 years to 33 years with 17 being female and 13 male. All subjects gave written informed consent in accordance with the Declaration of Helsinki and were paid €8 per hour or received course credit. In accordance with the responsible funding agency guidelines (http://www.dfg.de/foerderung/faq/geistes_sozialwissenschaften/), this study did not require formal approval by the institutional review board as the study was not associated with a risk of physical or emotional harm and did not involve clinical intervention or examination and also did not involve underaged, or elderly participants.

### Experimental Procedure

#### S-R-O Acquisition Phase

**Figure 1:**
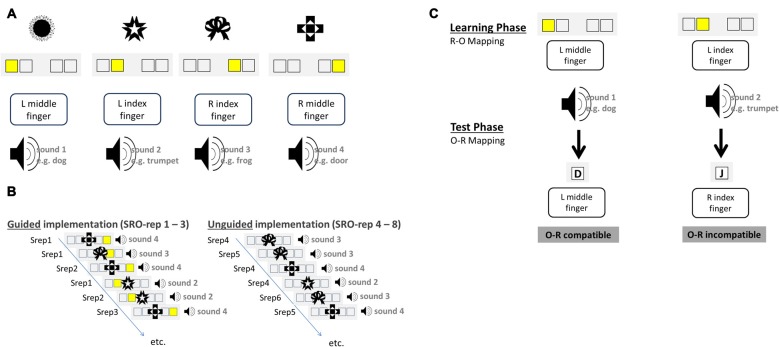
**Schematic illustration of the S-R-O acquisition phase (A,B)** and the O-R test phase **(C)** for an exemplary set of visual antecedent stimuli and auditory differential outcomes. Over the course of the experiment subjects had to learn 10 such S-R-O mappings each comprising a novel and distinct set of four visual stimuli and four auditory outcomes. **(A)** Exemplary mapping between visual stimuli, instruction cues, responses and auditory outcomes. **(B)** Novel S-R-O mappings were learned in the guided implementation trials via explicit instruction cues presented for the first three repetitions of each distinct S-R-O triple (SRO-rep 1–3). Starting from SRO-repetition 4 onwards instruction cues were omitted and the correct response had to be retrieved from memory in the unguided implementation trials (SRO-rep 4–8). **(C)** The four sound stimuli that had been produced by correct responses upon the antecedent visual stimuli in the learning phase served as antecedent stimuli in the test phase. The required responses to these stimuli could be the same (compatible) or different (incompatible) regarding the response that produced the sound before. Test phase data were exclusively used to compute the size of the behavioral O-R compatibility effect, which was correlated with learning-related changes in brain-electrical activity during the preceding S-R-O learning phase.

Instructions were delivered via a “guided implementation” procedure in which the instruction is embedded within the first three behavioral implementation trials that also comprised the presentation of differential outcomes following correct responses (see Figures 1A,B).

Stimuli were four abstract visual patterns that differed for each block. In the acquisition phase each trial started with the presentation of a visual stimulus S in the center of the screen for 500 ms. Each stimulus was presented at least eight times and additional presentations occurred for each erroneous response. Stimuli were presented in random order except post-error trials in which the same stimulus was presented again. There were 12 guided implementation trials (three repetitions of four different stimuli, correct responses and outcomes). Following 250 ms after S onset an additional instruction stimulus (IS) was displayed which remained on screen until a response was made or until timeout after 1750 ms. The IS was a yellow square highlighting one of four constantly displayed empty boxes. Manual responses (left middle finger, left index finger, right index finger and right middle finger) were mapped in a spatially compatible manner to the IS position. After a 150 ms gap, correct responses were followed by a naturalistic sound effect which lasted for 500 ms and was different for each S. In unguided implementation trials the IS was omitted for another five correct repetitions of the four distinct S-R-O triples. Thus, starting from the fourth S-R-O repetition (SRO-rep), the correct response had to be retrieved from memory as it was no longer indicated by the IS. In case of erroneous responses, error feedback was displayed and the trial was immediately repeated. The experiment comprised 10 different S-R-O learning blocks each with novel visual stimuli and novel outcome sounds. The inter-trial interval (ITI) was randomly selected from a distribution including interval durations of 800 ms (24 trials per block), 2350 ms (5 trials per block) and 4700 ms (3 trials per block). Analyses of learning-related changes in behavior and brain activation were based on correct trials ranging from SRO-rep 1 to SRO-rep 8.

#### O-R Test Phase

Each of the 10 S-R-O learning blocks was followed by a test phase probing the strength of the previously acquired bidirectional R-O associations. Subjects were now required to react to the previous effect sounds of the acquisition phase with one out of four responses (see Figure 1C). The response keys were the same as during the preceding S-R-O learning phase. Two outcome sounds were mapped to the response that produced that sound in the preceding phase (compatible trials) whereas the two remaining outcome sounds were mapped to responses that had produced another sound (incompatible trials). According to ideomotor theory, previously learnt bidirectional R-O associations should prime the correct response in compatible trials but the incorrect response in incompatible trials. The R-O compatibility effect was defined as the performance difference between incompatible and compatible trials averaged across all test phase trials separately for RTs and error rates. As in the preceding S-R-O acquisition phase, also the test phase was divided into 12 guided and 20 unguided trials. The instruction stimuli (IS) were now the letters “D”, “F”, “J”, “K” presented centrally on the screen and mapped onto left middle finger, left index finger, right index finger and right middle finger, respectively. A trial started with a fixation cross displayed for 500 ms followed by the sound lasting 500 ms. In the guided trials the IS was presented 150 ms after sound onset and lasted until the response or timeout after 1500 ms. Accuracy feedback was displayed for 650 ms indicating correct, wrong, or too slow responses. The ITI distribution was the same as in the preceding S-R-O learning phase.

### EEG Recording

EEG was recorded using 64 sintered Ag/AgCl electrodes which were distributed on the electrode cap (Easycap) according to the international 10% system (Klem et al., 1999). The FCz electrode served as reference (re-used after offline average referencing) and the AFz electrode served as ground. Impedance at each electrode was kept below 5 kΩ. Two additional electrodes were used to record vertical and horizontal eye movements. One was placed under the lower eye lid and the other 1 cm lateral to the right eye. EEG data were digitized using a 64 channel BrainAmp amplifier^1^ with a sampling rate of 1000 Hz and recorded with the BrainVision recorder software^1^.

### EEG Preprocessing

All EEG data was preprocessed using the Fieldtrip software downloaded October 2016 (Oostenveld et al., 2011). The continuous EEG data were segmented into epochs starting 250 ms prior to stimulus onset and ending 2000 ms after stimulus onset. The segmented data were highpass-filtered at 0.2 Hz and lowpass-filtered at 30 Hz. All channels were used to compute and apply an average-based new reference. Ocular and other obvious artifacts were corrected by excluding the respective independent components as they were visually identified based on topography and waveform after an independent component analysis was run within Fieldtrip (using the default parameters and estimating 63 independent components according to the number of electrodes). After preprocessing, the EEG data were further segmented to create response-locked epochs. Response-locked epochs were ranging from 350 ms pre-response to 450 ms post-response (i.e., 300 ms post-outcome onset). No baseline-correction was applied to the segmented EEG data.

### ERP Analysis

Response-locked and stimulus-locked epochs of EEG activity were averaged for each SRO repetition containing 40 correct trials per SRO repetition in each subject. For the analysis of very rapid learning dynamics associated with initial R-O encoding processes, we assessed ERP amplitude changes from SRO-rep 1 to SRO-rep 2 (defined by the slope of a linear regression line, i.e., SRO-rep 2 minus SRO-rep 1). For the analysis of slower learning dynamics associated with more gradual changes in association strength, we determined ERP amplitude changes from SRO-rep 2 through SRO-rep 8 based on the slope of a linear regression line fitted through the mean amplitude values of all seven involved SRO-rep levels.

The primary aim of the present study was to identify learning-related ERP changes on both timescales that were specifically linked to the learning of bidirectional R-O associations. To this end, we determined correlations between learning-related ERP amplitude changes and—as a proxy for the acquired bi-directional R-O association strength—the behavioral O-R compatibility effect from the test phase.

Our hypotheses regarding learning-related modulations of ERP amplitudes were based on previous ERP studies that differed from the present study in a number of potentially relevant aspects. First and foremost, previous ERP studies did *not* assess learning-related modulations during the initial phase of R-O learning. Moreover, previous studies examined ERP modulations in free-choice settings rather than in forced-choice settings. Finally, action-induced sounds were typically simple sine-wave tones with short durations around 100 ms rather than more complex natural sounds used in the present study which were developing across intervals of 300–500 ms. To be unbiased regarding possible learning-related ERP modulations in other than those expected according to related previous studies, we conducted statistical analyses across all electrodes and across relatively large time windows. The necessary correction for multiple comparisons (time points and electrodes) was based on the cluster-extent thresholding procedure employed in the fieldtrip software (Oostenveld et al., 2011).

We analyzed three time windows according to our hypotheses. First, to assess potential learning-related increases in action-induced sensory attenuation effects, we analyzed correlations between the O-R compatibility effect and response-locked ERPs in a time window ranging from the onset of the manual response until 450 ms later. Since the sound started 150 ms after response onset this time window covered both potential preparatory effects prior to sound onset and the typical attenuation effects following sound onset. Second, to assess potential learning-related amplifications of the readiness potential, we analyzed correlations between the O-R compatibility effect and response-locked ERPs in a time window starting 350 ms prior to the onset of the manual response until response onset. Third, to assess potential learning-related changes of stimulus-triggered outcome integration processes, we analyzed correlations between the O-R compatibility effect and stimulus-locked ERP in a time window ranging from the onset of the visual stimulus until 300 ms later. This time window was limited at 300 ms to ensure that ERPs would reflect stimulus-related modulations without being considerably contaminated by neural activity induced by the additional instruction cue that was presented 250 ms after the onset of the visual stimulus.

The analysis of correlations between the behavioral O-R compatibility effect and learning-related ERP modulations was conducted for each time window separately and covered all electrodes and all time points within each time window. Family-wise alpha was controlled at *p* < 0.05 (two-tailed) based on cluster-extent thresholding in time and space (requiring a minimum of one connection between electrodes) as implemented in the Fieldtrip software and using Monte Carlo simulations based on 5000 permutations.

### ERP Analysis: Controlling for Potential Confounding Factors

We took several measures to minimize the potential influence of confounding sources of covariance between learning-related ERP modulations and the behavioral O-R compatibility effect. Recall that the study rationale rests on the assumption that inter-individual differences in R-O learning during the SRO learning phase (probed by ERP changes) should result in different R-O association strengths (probed by the O-R compatibility effect in the subsequent test phase). However, the correlation between the O-R compatibility effect and preceding learning-related ERP modulations could be confounded by two alternative source of co-variation: one related to inter-individual differences in competition resolution ability and the other one related to inter-individual differences in general learning ability.

First, inter-individual differences in competition resolution ability might not only affect the size of the O-R compatibility effect due to increased competition resolution demands in incompatible vs. compatible test phase trials, but it might similarly affect learning-related ERP modulations during the preceding SRO learning phase that are related to changing demands of resolving competition between the correct response for a given stimulus and the three alternative (but wrong) response options.

Second, inter-individual differences in general learning ability might not only affect learning processes expressed in learning-related ERP modulations during the SRO learning phase, but it might similarly affect learning processes occurring within the test phase which might influence the O-R compatibility effect^2^. Specifically, such test phase learning processes might be related to the re-wiring or re-learning of the old bi-directional R-O mapping according to the newly instructed mapping between the former outcomes (now serving as antecedent stimuli) and the new test phase responses. This has two potential implications. The first implication is that even though a good learner enters the test phase with strong bidirectional R-O associations this might nevertheless result in a relatively weak overall O-R compatibility effect as the old bidirectional R-O mapping could be quickly re-wired. This in turn implied that larger learning-related ERP modulations due to stronger R-O learning would be associated with weaker O-R compatibility effects rather than stronger O-R compatibility effects. This ambiguity complicates the interpretation of the direction of the correlation between R-O-learning-related ERP modulations and the O-R compatibility effect. However, any significant correlation—no matter which direction—is suited to conclude that the identified learning-related ERP modulation is in one way or the other related to R-O learning. The second implication is potentially more problematic. Specifically, inter-individual differences in general learning ability might not only affect ERP modulations related to O-R learning but it might also affect ERP modulations related to S-R learning. Hence, a significant correlation between O-R compatibility effect and learning-related ERP modulation might be due to co-variation between R-O/O-R re-learning ability in the test phase and S-R learning ability (instead of R-O learning ability) in the preceding SRO learning phase.

Fortunately, inter-individual differences in S-R learning ability and inter-individual differences in competition resolution ability as two potential cofounding factors are reflected by similar behavioral measures and can therefore be controlled for in a very similar manner. Specifically, inter-individual differences in these two abilities should be expressed in differences in learning-related changes in error rates and response times. For instance, a strong reduction in error rates could be due to high S-R learning ability, high competition resolution ability, or a combination of both. Accordingly, before computing correlations between the O-R compatibility effect and learning-related ERP changes, we regressed out learning-related changes in error rates and response times (corresponded to the specific ERP change being evaluated) as proxies for changes in response competition/progress in S-R learning during the SRO learning phase. A related measure was taken as special treatment regarding the transition from the guided learning phase (instruction cue present) to the unguided learning phase (instruction cue absent). This abrupt transition might imply changes in response competition/progress in S-R learning that are not sufficiently captured by corresponding changes in RTs and error rates. We therefore performed an additional control analysis regarding learning on the slower timescale (i.e., linear ERP change from SRP-rep 2 to SRO-rep 8) where we separately assessed the transition from guided to unguided SRO repetitions (linear regression line fitted through the mean amplitude values across SRO-rep 2 through SRO-rep 4) and learning dynamics across *unguided* SRO repetitions only (linear regression line fitted through the mean amplitude values across SRO-rep 4 through SRO-rep 8).

## Results

### Behavioral Results (S-R-O Learning Phase)

**Figure 2:**
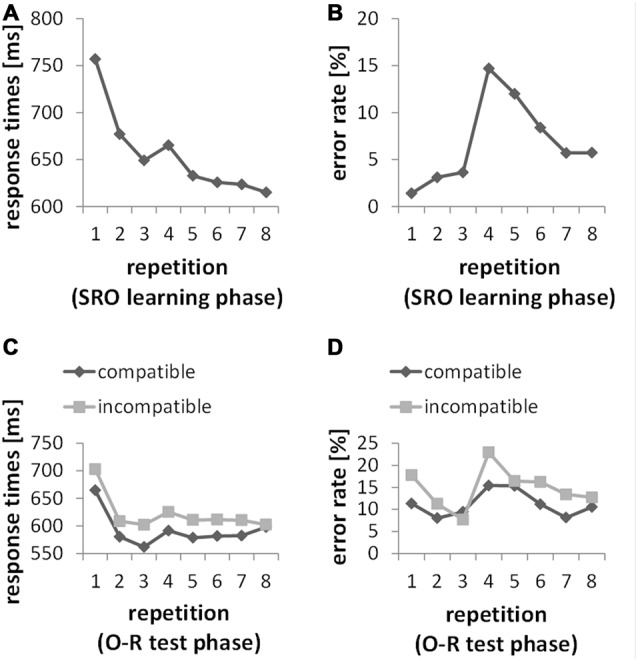
**(A,B)** Behavioral data from the S-R-O acquisition phase. Responses were guided by a spatial instruction cue during SRO repetitions 1 through 3. Starting from SRO repetition 4 up to 8, instruction cues were no longer presented. **(C,D)** Behavioral data from the O-R test phase. Responses were guided by a symbolic instruction cue during O-R repetitions 1 through 3. Starting from O-R repetition 4 up to 8, instruction cues were no longer presented.

Figure 2 depicts the learning curves across all SRO repetitions in RTs and error rates. The statistical analysis (based on repeated measures ANOVAs with the factor SRO repetition) confirmed the key features of the two learning profiles as they appear on the descriptive level. Generally, increasing SRO repetitions were associated with an overall drop of response times (*F*_(7,203)_ = 59.98; *p*_(F)_ < 0.001; $\eta_{\text{p}}^{\text{2}}$ = 0.674) paralleled by an increase in error rates (*F*_(7,203)_ = 23.21; *p*_(F)_ < 0.001; $\eta_{\text{p}}^{\text{2}}$ = 0.445). The guided learning trials (SRO-rep 1 to SRO-rep 3) were characterized by a rapid gain in performance speed (*F*_(2,58)_ = 115.43; *p*_(F)_ < 0.001; $\eta_{\text{p}}^{\text{2}}$ = 0.799). At SRO-rep 4 (i.e., the first unguided trials) RT briefly increased likely due to the transition to the unguided phase as indicated by a *t-test* comparing SRO-rep 4 against SRO-rep 3 (*t*_(29)_ = 2.08; *p*_(t)_ < 0.046, two-tailed) before RTs continued to decrease gradually across repetitions 4–8 (*F*_(4,116)_ = 10.97; *p*_(F)_ < 0.001; $\eta_{\text{p}}^{\text{2}}$ = 0.274). Unsurprisingly, error rate was low during the guided trials with a slight but significant increase (*F*_(2,58)_ = 4.47; *p*_(F)_ < 0.017; $\eta_{\text{p}}^{\text{2}}$ = 0.134). At SRO-rep 4 (i.e., the first unguided repetition) error rate abruptly jumped up as indicated by a *t*-test comparing SRO-rep 4 against SRO-rep 3 (*t*_(29)_ = 5.43; *p*_(t)_ < 0.001) before gradually decreasing again across the unguided repetitions 4–8 (*F*_(4,116)_ = 16.89; *p*_(F)_ < 0.001; $\eta_{\text{p}}^{\text{2}}$ = 0.368).

### Behavioral Results (O-R Test Phase)

We computed two separate repeated-measures ANOVAs for error rates and RTs with the factors O-R compatibility and O-R repetitions (i.e., repeated pairings of the former O—now serving as an antecedent S—with its test phase response). The analysis for error rates resulted in a highly significant O-R compatibility effect (*F*_(1,29)_ = 21.39, *p* < 0.001; $\eta_{\text{p}}^{\text{2}}$ = 0.425) with an average of 11.2% errors in the compatible condition and 14.8% errors in the incompatible condition. The analysis for RTs resulted in a highly significant O-R compatibility effect (*F*_(1,29)_ = 18.81, *p* < 0.001; $\eta_{\text{p}}^{\text{2}}$ = 0.393) with an average of 592 ms in the compatible condition and 622 ms in the incompatible condition. Additionally, the interaction between O-R compatibility and O-R repetitions in error rates just reached significance (*F*_(7,203)_ = 2.27, *p* = 0.048, Greenhouse-Geisser-corrected). However, this interaction followed a rather complex and non-monotonic pattern (primarily driven by a 7th order polynomial contrast effect with *p* < 0.007) which seems difficult to interpret in terms of incremental learning-related changes. Regarding response times no such interaction effect was found (*F*_(7,203)_ = 0.72, *p* = 0.61).

The subject-wise behavioral index of R-O associational strength which was later used for correlation with ERP difference measures was defined as the individual mean O-R compatibility effect in RTs, providing a more consistent measure across all O-R repetitions compared to the compatibility effect in error rates. Moreover, the observation that the test phase O-R compatibility effect in RTs did *not* significantly decrease across O-R repetitions suggests that this measure is *not* strongly affected by a re-wiring of previously established bi-directional R-O associations. Rather, the previously established R-O associations seem to co-exist with the newly instructed associations between the former O (now S) and the newly instructed test phase responses at least for the duration of the test phase. Otherwise, an increasing number of test phase learning trials should have resulted in stronger re-wiring and hence an increasingly weaker O-R compatibility effect. In turn, this suggests that inter-individual variability in the size of the overall O-R compatibility effect predominantly reflects inter-individual differences in the size of an enduring response selection bias exerted by the previously established bidirectional R-O associations rather than reflecting inter-individual differences in (re-) learning ability. Furthermore, the O-R compatibility effect did not significantly correlate with learning-related behavioral measures in the preceding SRO learning phase. Specifically, there were no significant correlations (all *p*_(r)_ > 0.49) with RT or error rate change both regarding the rapid timescale (SRO-rep 1 vs. SRO-rep2) and the slower timescale (SRO-rep 2 vs. SRO-rep 8). Note that previous findings were ambiguous in this respect. While we found significant correlations between the O-R compatibility effect and learning-related RT change in one study (Ruge et al., 2012), this could not be replicated in a subsequent study (Ruge and Wolfensteller, 2015). None of these previous studies reported significant correlations with learning-related changes in error rates. Hence, together these results suggest a rather unreliable association between the O-R compatibility effect and learning-related behavioral measures.

### EEG Results

#### Response-Locked ERPs—Post-Response Modulations

**Figure 3:**
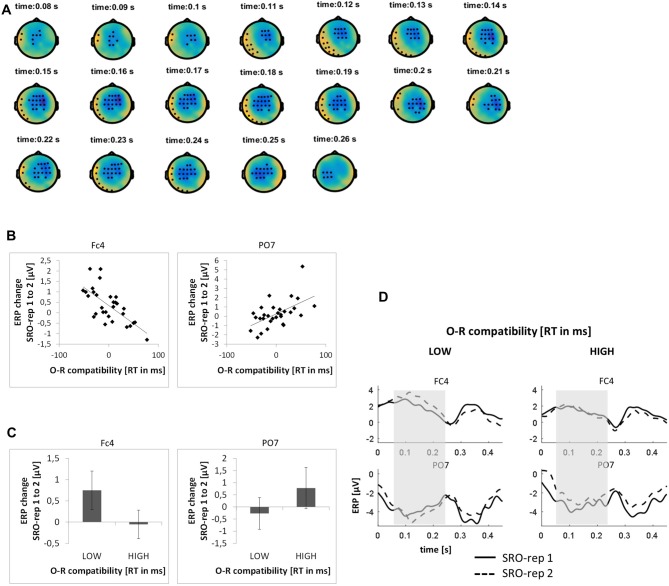
**Relationship between learning-related response-locked event-related potential (ERP) changes on the rapid timescale (i.e., change from SRO-rep 1 to SRO-rep 2) and the behavioral O-R compatibility effect. (A)** Significant spatio-temporal clusters (marked by black dots) for the correlation between ERP changes and O-R compatibility. **(B)** Exemplary scatter plots for the electrodes with the strongest correlation effects averaged across all timepoints within the respective spatiotemporal cluster. **(C)** Median-split representation of the scatter plot data. **(D)** Median-split representation of the continuous ERP timecourses for peak electrodes within the respective clusters. Shaded areas denote the approximate time window of significant correlation effects.

This analysis was based on a time window from 0 ms to 450 ms relative to response onset (i.e., −150 ms to 300 ms relative to sound outcome onset). On the rapid timescale, we identified two spatiotemporal clusters which exhibited significant correlations between the ERP amplitude change from SRO-rep 1 to SRO-rep 2 and the behavioral O-R compatibility effect (see Figure 3A): a fronto-centrally distributed cluster exhibited a negative correlation in a time window from 79 ms to 262 ms (*p* < 0.0006, FWE-corrected on cluster level) and a left lateralized cluster including fronto-temporal and parieto-temporal electrodes exhibited a positive correlation in a time window from 42 ms to 244 ms (*p* < 0.016, FWE-corrected on cluster level). Figure 3B summarizes these correlations by means of scatter plots for the respective peak electrodes and averaged across all significant time points within each cluster. Table 1 additionally reports these time-average-based correlations in numbers and shows that the effects observed at the rapid timescale did not generalize to the slower timescale. To better assess the direction of amplitude changes as a function of the O-R compatibility effect, Figures 3C,D depict mean ERP amplitude changes after a median-split according to the size of the O-R compatibility effect. This representation of the original results suggests that subjects exhibiting a large O-R compatibility effect show a reduced learning-related increase in the positivity at FC4 (paralleled by a reversed effect at PO7) relative to the subjects exhibiting a small O-R compatibility effect. Note that even though this effect extended into the time window of the auditory N1 component, it is clearly not related to the standard sensory attenuation effect. In fact, the sensory attenuation effect is characterized by a reduced negativity within the N1 latency range whereas the present effect was pointing in exactly the opposite direction (i.e., a relatively *increased* negativity or, in other words, a relatively reduced positivity).

**Table 1:** **Summary of correlations between the O-R compatibility effect and response-locked event-related potential (ERP) amplitude changes at the respective peak electrodes within each identified cluster**.

		ERP change from SRO-rep 1 to 2	ERP change from SRO-rep 2 to 8	ERP change from SRO-rep 2 to 4	ERP change from SRO-rep 4 to 8
Clusters identified on the very rapid timescale (ERP change from SRO-rep 1 to 2)	Frontocentral (peak FC4)	***r* = −0.68**	*r* = 0.2	NA	NA
	79 –262 ms	***p*_(r)_ < 0.00003**	*p*_(r)_ < 0.30 (n.s.)
	Posterior (peak PO7)	***r* = 0.56**	*r* = −0.12	NA	NA
	42 –244 ms	***p*_(r)_ < 0.0014**	*p*_(r)_ < 0.53 (n.s.)	NA	NA
Clusters identified on the slower timescale (ERP change from SRO-rep 2 to 8)	Frontocentral (peak Fz)	*r* = −0.23	***r* = 0.62**	*r* = 0.27	***r* = 0.38**
	327 –436 ms	*p*_(r)_ < 0.23 (n.s.)	***p*_(r)_ < 0.0003**	*p*_(r)_ < 0.15 (n.s.)	***p*_(r)_ < 0.037**
	Posterior (peak PO7)	*r* = 0.08	***r* = −0.55**	*r* = 0.01	***r* = −0.44**
	312 –450 ms	*p*_(r)_ < 0.67 (n.s.)	***p*_(r)_ < 0.002**	*p*_(r)_ < 0.97 (n.s.)	***p*_(r)_ < 0.015**

*Bold font indicates significant correlations*.

**Figure 4:**
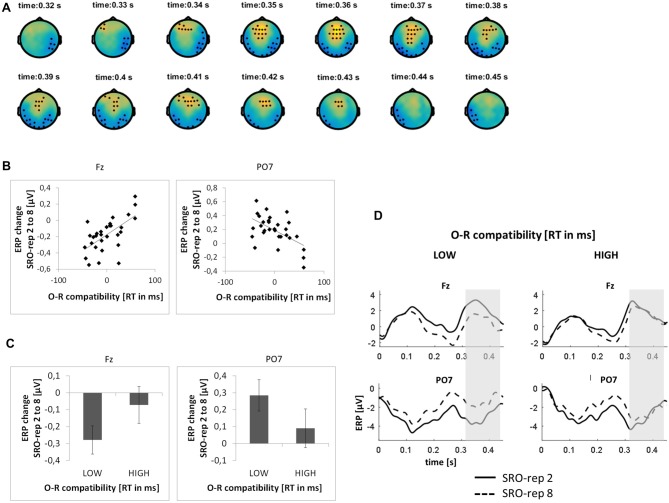
**Relationship between learning-related response-locked ERP changes on the slower timescale (i.e., linear change from SRO-rep 2 to SRO-rep 8) and the behavioral O-R compatibility effect. (A)** Significant spatio-temporal clusters (marked by black dots) for the correlation between ERP changes and O-R compatibility. **(B)** Exemplary scatter plots for the electrodes with the strongest correlation effects averaged across all timepoints within the respective spatiotemporal cluster. **(C)** Median-split representation of the scatter plot data. **(D)** Median-split representation of the continuous ERP timecourses for peak electrodes within the respective clusters. Shaded areas denote the approximate time window of significant correlation effects.

On the slower timescale, we identified two spatiotemporal clusters which exhibited significant correlations between the linear ERP amplitude change from SRO-rep 2 to SRO-rep 8 and the behavioral O-R compatibility effect (see Figure 4A): a fronto-centrally distributed cluster exhibited a positive correlation in a time window from 327 ms to 436 ms (*p* < 0.047, FWE-corrected on cluster level) and a posteriorly distributed cluster exhibited a negative correlation in a time window from 312 ms to 450 ms (*p* < 0.042, FWE-corrected on cluster level). Figure 4B summarizes these correlations by means of scatter plots for the respective peak electrodes and averaged across all significant time points within each cluster. Table 1 additionally reports these time-average-based correlations in numbers and shows that the effects observed at the slower timescale did not generalize to the rapid timescale. To better assess the direction of amplitude changes as a function of the O-R compatibility effect, Figures 4C,D depict mean ERP amplitude changes after a median-split according to the size of the O-R compatibility effect. This representation of the original results clearly suggests that subjects exhibiting a large O-R compatibility effect show an attenuated learning-related increase in the negativity at Fz (paralleled by a reversed effect at PO7) relative to the subjects exhibiting a small O-R compatibility effect.

To exclude that these effects were solely driven by the transition from the guided trials to the unguided trials, we separately assessed the correlations between the O-R compatibility effect and the linear ERP amplitude change from SRO-rep 2 to SRO-rep 4 (transition guided to unguided) as well as the linear ERP amplitude change from SRO-rep 4 to SRO-rep 8 (across unguided trials). As summarized in Table 1, the only significant correlations were observed for the ERP amplitude changes across the unguided trials. This refutes the possibility that the original correlational effects were primarily driven by the guided-to-unguided transition.

Finally, to test whether the correlational effects observed on the very rapid timescale and on the slower timescale were driven by similar sources of co-variance across participants, we computed partial correlations. This revealed that the two original correlation effects were largely independent of each other, as detailed next. First, we computed for the anterior clusters the correlation between *slower* learning-related ERP changes and the O-R compatibility effect while controlling for *rapid* learning-related ERP changes. This yielded a correlation of *r* = 0.54 (*p* < 0.002) which was only marginally weaker than the original correlation (*r* = 0.62, see Table 1). Second, we computed for the anterior clusters the correlation between *rapid* learning-related ERP changes and the O-R compatibility effect while controlling for *slower* learning-related ERP changes. This yielded a correlation of *r* = −0.62 (*p* < 0.0003) which was again only marginally weaker than the original correlation (*r* = −0.68, see Table 1). A very similar pattern was found for the posterior clusters (*r* = −0.48 vs. *r* = −0.55 originally and *r* = 0.50 vs. *r* = 0.56 originally).

#### Response-Locked ERPs—Pre-Response Modulations

This analysis was based on a time window from −350 ms to 0 ms relative to response onset. There were no significant effects, both on the rapid timescale as well as on the slower timescale. We repeated these analyses, this time only including the Cz electrode where previous studies reported readiness potential-like potentials in conditions with stronger involvement of bidirectional R-O associations. Again, there were no significant effects.

#### Stimulus-Locked ERPs

**Figure 5:**
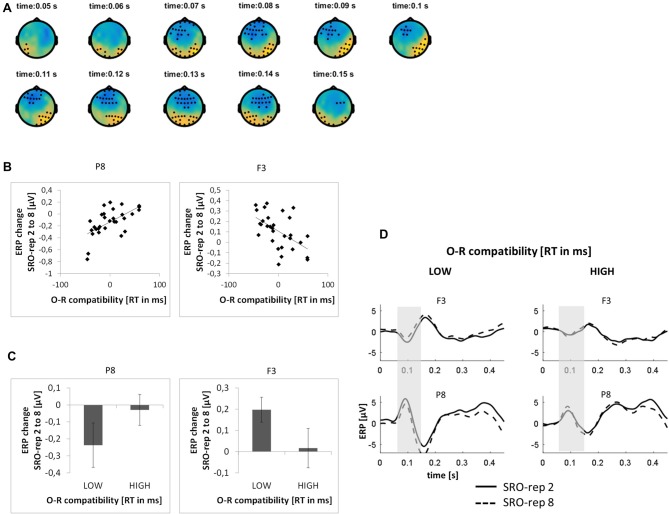
**Relationship between learning-related stimulus-locked ERP changes on the slower timescale (i.e., linear change from SRO-rep 2 to SRO-rep 8) and the behavioral O-R compatibility effect. (A)** Significant spatio-temporal clusters (marked by black dots) for the correlation between ERP changes and O-R compatibility. **(B)** Exemplary scatter plots for the electrodes with the strongest correlation effects averaged across all timepoints within the respective spatiotemporal cluster. **(C)** Median-split representation of the scatter plot data. **(D)** Median-split representation of the continuous ERP timecourses for peak electrodes within the respective clusters. Shaded areas denote the approximate time window of significant correlation effects.

This analysis was based on a time window from 0 ms to 300 ms relative to response onset. On the rapid timescale, we did not find significant effects. On the slower timescale, we identified two spatiotemporal clusters which exhibited significant correlations between the linear ERP amplitude trend from SRO-rep 2 to SRO-rep 8 and the behavioral O-R compatibility effect (see Figure 5A): a fronto-centrally distributed cluster exhibited a negative correlation in a time window from 64 ms to 150 ms (*p* < 0.013, FWE-corrected on cluster level) and a posteriorly distributed cluster exhibited a positive correlation in a in a time window from 45 ms to 159 ms (*p* < 0.007, FWE-corrected on cluster level). Figure 5B summarizes these correlations by means of scatter plots for the respective peak electrodes and averaged across all significant time points within each cluster. Table 2 also reports these average-based correlations in numbers and shows that the effects observed at the slower timescale did not generalize to the rapid timescale. To better assess the direction of amplitude changes as function of the O-R compatibility effect, Figures 5C,D depict mean ERP amplitude changes after a median-split according to the size of the O-R compatibility effect. This representation of the original results suggests that subjects exhibiting a large O-R compatibility effect show an attenuated learning-related increase in the negativity at P8 (paralleled by a reversed effect at F3) relative to the subjects exhibiting a small O-R compatibility effect.

**Table 2:** **Summary of correlations between the O-R compatibility effect and stimulus-locked ERP amplitude changes at the respective peak electrodes within each identified cluster**.

		ERP change from SRO-rep 1 to 2	ERP change from SRO-rep 2 to 8	ERP change from SRO-rep 2 to 4	ERP change from SRO-rep 4 to 8
Clusters identified on the very rapid timescale (ERP change from SRO-rep 1 to 2)	None	NA	NA	NA	NA
Clusters identified on the slower timescale (ERP change from SRO-rep 2 to 8)	Frontocentral (peak F3)	*r* = 0.29	***r* = −0.55**	***r* = −0.33**	*r* = −0.28
	64 –150 ms	*p*_(r)_ < 0.12 (n.s.)	***p*_(r)_ < 0.002**	*p*_(r)_ < 0.072*	*p*_(r)_ < 0.13 (n.s.)
	Posterior (peak P8)	*r* = 0.21	***r* = 0.60**	***r* = 0.48**	*r* = 0.35
	45 –159 ms	*p*_(r)_ < 0.26 (n.s.)	***p*_(r)_ < 0.0004**	***p*_(r)_ < 0.007**	*p*_(r)_ < 0.061*

*Significant when tested one-tailed in the direction of the correlation for SRO-rep 2 to 8. Bold font indicates significant correlations.

To exclude that these effects were solely driven by the transition from the guided trials to the unguided trials, we separately assessed the correlations between the O-R compatibility effect and the linear ERP amplitude change from SRO-rep 2 to SRO-rep 4 (transition guided to unguided) as well as the linear ERP amplitude change from SRO-rep 4 to SRO-rep 8 (across unguided trials). As summarized in Table 2, the pattern was not as clear as for the response-locked data in terms of statistical significance. Yet, numerically, the correlations for the fronto-central cluster were quite similar for the ERP amplitude changes across guided and unguided SRO repetitions (*r* = −0.33) and within unguided repetitions (*r* = −0.28), suggesting that the original correlation (*r* = −0.55) was driven more or less equally by both. The same holds for the posterior cluster. Hence, in summary, this again refutes the possibility that the original correlational effects were primarily driven by the guided-to-unguided transition.

## Discussion

This study was designed to investigate ERP dynamics associated with the initial learning of bi-directional R-O associations under forced choice conditions by assessing correlations with the post-learning behavioral O-R compatibility effect as an index of the previously acquired association strength. This goes beyond earlier EEG studies (for review see Waszak et al., 2012) and fMRI studies (Kühn et al., 2010; Ruge et al., 2010; Pfister et al., 2014; Zwosta et al., 2015) which have revealed neurophysiological markers related to the impact of learnt R-O associations but which have not yet investigated the initial learning of such associations. Moreover, by assessing ERPs we could easily disentangle *within* trials the potential pre-response and post-response activation of bidirectional R-O associations as they were being learned. Due to the sluggish nature of the BOLD response this was difficult to assess in previous fMRI which have begun to characterize the evolution of R-O integration processes *across* learning trials on multiple time scales of learning (Melcher et al., 2013; Ruge and Wolfensteller, 2013, 2015; Mohr et al., 2015).

Generally, we found that the O-R compatibility effect was significantly associated with both post-response as well as pre-response ERP modulations that can therefore be linked to the initial learning of bi-directional R-O associations. Some of these effects were observed on a very rapid timescale from the first to the second repetition of specific R-O pairings while others were observed on a slower timescale from the second to the final eighth R-O repetition. Importantly, these results were found after controlling for a number of potential confounding sources of covariation between learning-related ERP modulations and the O-R compatibility effect, including inter-individual differences in general learning ability and inter-individual differences in competition resolution ability (see “ERP Analysis” Section).

As a general note, it should be highlighted that our correlational approach added an additional layer of ambiguity to the interpretation of the direction of the correlated ERP modulations. Even for simple ERP difference measures, it is difficult to know whether the ERP amplitude modulation was due to increasing strength of one dipole or the decreasing strength of another dipole with inverse polarity (Luck, 2005). If ERP modulations are identified via correlations with an external measure, it becomes even more important to be aware of the fact that the direction of these correlations does not directly and unambiguously translate into the direction of the underlying change in dipole strength.

### Post-Response ERP Modulations

Previous EEG studies have demonstrated post-response reductions of fronto-central ERP amplitudes following the onset of auditory action outcomes within the auditory N1-P2 range. This has typically been interpreted as a likely marker of action-induced sensory attenuation effects due to previously acquired bi-directional R-O associations. Our results suggest that such attenuation effects can be established within very few repetitions of specific R-O pairings (i.e., across repetitions 2–8). Moreover, our results highlight that such attenuation effects generalize to situations in which response selection is based on an antecedent stimulus (i.e., forced choice) in contrast to “voluntary” or free choice response selection realized in previous studies. This is likely due to the circumstance that stimulus-based response selection is still intentional in nature given the limited time for habitualization within only eight S-R-O repetitions in the particular study design we employed (Wolfensteller and Ruge, 2012; Ruge and Wolfensteller, 2013).

In the present study, following the onset of auditory outcomes 150 ms after response execution, the attenuation effect was expressed by a stronger relative reduction of an anterior negativity across learning trials for subjects exhibiting a stronger O-R compatibility effect as a proxy for acquired R-O associational strength. Notably, this learning-related attenuation effect was embedded within an overall mean *increase* of the anterior negativity. This overall learning-related increase of the anterior negativity, however, was likely due to a superimposed ERP modulation associated with perceptual learning based on the mere repetition of auditory outcomes which is unrelated to R-O learning (Atienza et al., 2002; Alain et al., 2007; Mishra et al., 2015).

Interestingly, the present attenuation effect was maximal clearly after the auditory N1 peak. Even though a number of previous studies observed maximal effects of action-induced sensory attenuation after the auditory N1 peak (Baess et al., 2011; SanMiguel et al., 2013), it was shifted even further in our paradigm. This is most likely due to the different nature of the presented auditory stimuli. Previous studies typically used pure sine tones with short durations (50–140 ms) whereas we used relatively complex natural sounds with a rather long duration of 300–500 ms. This might imply longer perceptual analysis times which might translate into rather late and longer lasting ERP attenuation effects. Support for this possibility comes from a study which analyzed self-generated speech sounds (i.e., more complex stimuli) and found longer lasting attenuation effects (Houde et al., 2002). By contrast, it seems rather unlikely that the observed temporal shift was due to the relatively long 150 ms interval between response execution and outcome onset as a few earlier studies suggested that the attenuation effect was unaffected by response-outcome delays of up to 1000 ms, regardless of whether the onset of outcome presentation was predictable or not (Baess et al., 2011; Lange, 2011; SanMiguel et al., 2013). Finally it should be noted that the fronto-central ERP effect was accompanied by a reversed polarity effect at posterior electrodes. This is in line with a previous study which suggested that such a bipolar anterior-posterior topography of the action-induced sensory attenuation effect might be specific for highly predictable onsets as in the present study (Baess et al., 2011).

In addition to the hypothesized learning-related ERP attenuation effect triggered by outcome presentation 150 ms after response execution, we also observed a post-response but *pre-outcome* ERP modulation. Specifically, we found that subjects exhibiting a strong O-R compatibility effect exhibited a strongly reduced learning-related positivity that was otherwise evolving very rapidly from SRO-rep 1 to SRO-rep 2 for subjects exhibiting a weak O-R compatibility effect. That this ERP effect started well before outcome onset suggests that it is related to the rapid deployment of preparatory attention towards expected outcomes rather than altered outcome processing itself. Given that previous studies of preparatory attention found increased negativities being associated with the preparatory deployment of attention, we speculate that the reduced learning-related positivity was in fact due to a superimposed increased learning-related negativity for strong R-O learners. In any case, increased deployment of preparatory attention could be interpreted either as a pre-condition for superior R-O learning (reflecting heightened attention towards outcome sounds and hence better learning) or as consequence thereof (reflecting better R-O learning and hence heightened attention).

Notably, the rapid learning-related pre-outcome modulation of ERP amplitudes and the slower learning-related peri-outcome modulation of ERP amplitudes (i.e., the sensory attenuation effect), while exhibiting similar topographies, were still independent predictors of O-R compatibility as suggested by follow-up partial correlation analyses.

### Absence of Pre-Response, Response-Locked ERP Modulations

It has been suggested that intention-based action planning relies more heavily on the backward activation (via outcome anticipation) of bidirectional R-O associations under free choice conditions as compared to stimulus-based forced choice conditions (Krieghoff et al., 2011). Based on this assumption, previous ERP studies suggested that this is reflected by a more pronounced readiness potential-like component as a general marker of intention-based action planning (Waszak et al., 2005; Krieghoff et al., 2011). Nevertheless, we hypothesized that we might observe a similar ERP amplification even under forced choice conditions especially for good R-O learners. We reasoned that stimulus-based forced-choice response selection would still be intentional in nature as habitualization processes should not have considerably kicked in due to the limited number of S-R repetitions (Ruge et al., 2012; Ruge and Wolfensteller, 2013). Yet, we did not observe any such effect. On the one hand, this might suggest that stimulus-based response selection generally does not involve the anticipatory backward activation of bidirectional R-O associations—even in an early, pre-habitualization phase of learning. On the other hand, it might suggest that the previous findings were restricted to the specific procedural setup being used and that readiness-potential-like effects are not generally indicative of intentional action planning processes. These two extreme positions could be reconciled by conceding that the anticipatory backward activation of bidirectional R-O associations might in fact occur even under forced-choice conditions, yet without impacting the type of intentional action planning processes that have been suggested to be reflected by a more pronounced readiness-potential-like component in free-choice situations. Alternatively, such intentional action planning processes might in fact occur even under forced-choice conditions, but they might simply not be correlated with the O-R compatibility effect we used to identify ERP markers of such processes (see Ruge and Wolfensteller, 2015). This complicated relationship between the O-R compatibility effect and *pre-response* ERP markers of intention-based action planning processes during the preceding SRO learning phase is also mirrored by a rather elusive relationship between the O-R compatibility effect and potential behavioral measures of intention-based action planning during the SRO learning phase. On the one hand, in previous studies we found some indication that increasing intention-based action planning across S-R-O learning compared to S-R learning might be reflected by increasing relative response slowing (Ruge et al., 2012; Ruge and Wolfensteller, 2013), and this slowing was in fact even significantly correlated with the subsequent O-R compatibility effect (Ruge et al., 2012). This was taken as evidence for increasing integration of bidirectional R-O associations into action planning mediated via an S-O, O-R activation chain. On the other hand, these correlational findings could not be confirmed in the present study as well as in a previous study using the same paradigm (Ruge and Wolfensteller, 2015). One reason for these inconsistencies across studies might be related to the fact that overt behavioral performance during the SRO learning phase is predominantly determined by S-R learning due to the simplicity of the 4:4 S-R mapping being instructed. Hence, stimulus-triggered action selection might be completed before the S-O, O-R activation chain could take effect reliably. That the simplicity of 4:4 S-R mappings might be a relevant factor was already suggested by previous studies which failed to find evidence for faster reduction in error rates when comparing an S-R-O condition with a pure S-R condition both during trial-and-error learning (Ruge et al., 2012) as well as during instruction-based learning (Ruge and Wolfensteller, 2013). This absence of error rate effects is inconsistent with findings from the original differential outcomes paradigm which seems to produce such effects, but for considerably more difficult learning problems and/or special populations characterized by inferior learning abilities (Trapold and Overmier, 1972; Estévez et al., 2001; Mok and Overmier, 2007; Martínez et al., 2009). Cleary, more research is needed to further clarify this issue.

### Pre-Response Stimulus-Locked ERP Modulations

We found a relatively reduced stimulus-locked negativity across learning associated with stronger O-R compatibility effects within the visual P1-N1 range in response to the visual antecedent stimulus. Analogously to the post-response attenuation effect, the learning-related stimulus-locked ERP attenuation effect was embedded within an overall mean *increase* of the posterior negativity. Again, this suggests that the stimulus-locked ERP attenuation effect was superimposed onto a larger general learning-related *increase* of the visual N1 which is likely reflecting perceptual learning as a result of repeated presentations of visual stimuli alone (Clark et al., 2015; Mishra et al., 2015), especially with the visual material being rather complex (Song et al., 2005) and unfamiliar (Brem et al., 2005).

But why are such early visual stimulus-induced ERPs associated with outcome-related learning processes. We speculate that this might be due to the fact that visual stimuli and auditory outcomes belong to different perceptual modalities and therefore might involve neural markers typically associated with multi-sensory integration processes (Murray et al., 2016). Multi-sensory integration processes, that is, the interaction between stimulus processing in different sensory modalities have been identified in similarly early stages of stimulus processing by previous ERP studies (Fort et al., 2002; Molholm et al., 2004; Talsma et al., 2007). Importantly, in the present study the putative multi-sensory integration effect can only be due to concurrent stimulus perception and outcome anticipation due to S-O learning. Yet, the stimulus-locked attenuation effect was revealed by a significant correlation with a behavioral marker of bidirectional R-O learning. But how is this relationship between S-O learning and R-O learning mediated? One possibility is that outcome anticipation triggered by the visual stimulus via learned S-O associations will amplify the strengthening of bidirectional R-O associations. In other words, the earlier an outcome is anticipated (based on S-O) the better it will be associated with R (leading to stronger O-R compatibility effects later on). This account is consistent with the general notion that more salient events (here outcomes that are pre-activated sooner) will be associated with other events (here the action) more easily and more rapidly (Mackintosh, 1975; Le Pelley and McLaren, 2003).

## General Conclusions

This study set out to characterize ERP markers associated with the acquisition of bi-directional R-O associations in a forced choice setting where action selection could in principle rely on S-R associations alone. However, previous work suggested that in an early pre-habitualization phase of learning subjects adopt an intention-based action mode involving R-O associations even under forced choice conditions. In line with this interpretation, we found that initial R-O learning under forced choice conditions was reflected by an increasing attenuation of outcome-induced ERP deflections similar to those identified in previous studies for extensively practiced R-O associations under free choice conditions. Moreover, we found a rather elusive temporary ERP amplification from the first to the second R-O repetition which emerged after response execution but before the onset of outcome presentation possible indicating the initial attentional gating of relevant information (here: the outcome). Obviously, such rapidly developing learning dynamics can only be detected when they are analyzed and previous ERP studies simply did not employ paradigms that were well suited to access the R-O learning process itself. We did not, however, find any evidence for the amplification of a readiness-potential-like ERP modulation which has previously been described for extensively trained R-O associations specifically for free choice settings when compared to forced choice settings. To better understand these contrasting results future research will need to clarify possible interactions between the amount of R-O training and choice mode. In contrast to response-locked ERP modulations, we entered entirely uncharted territory regarding stimulus-locked ERP markers associated with R-O learning (simply because there was no antecedent stimulus in previous free choice studies). This analysis showed that initial R-O learning was reflected by an increasing attenuation of early stimulus-induced ERP deflections in the visual P1-N1 latency range which seems to be consistent with the multi-sensory integration of the perceived antecedent visual stimulus and the anticipated auditory outcome as a possible amplifier for R-O learning under forced choice conditions.

## Author Contributions

FB, HR and UW designed the experiment; wrote the manuscript. FB collected the data; FB and HR analyzed the data.

## Conflict of Interest Statement

The authors declare that the research was conducted in the absence of any commercial or financial relationships that could be construed as a potential conflict of interest.

## Footnotes

^1^www.brainproducts.com

^2^We thank one of the reviewers for pointing this out.

## References

[B1] Alain C. Snyder J. S. He Y. Reinke K. S. (2007). Changes in auditory cortex parallel rapid perceptual learning. Cereb. Cortex 17, 1074–1084. 10.1093/cercor/bhl018 16754653

[B2] Aliu S. O. Houde J. F. Nagarajan S. S. (2009). Motor-induced suppression of the auditory cortex. J. Cogn. Neurosci. 21, 791–802. 10.1162/jocn.2009.21055 18593265 PMC2944400

[B3] Atienza M. Cantero J. L. Dominguez-Marin E. (2002). The time course of neural changes underlying auditory perceptual learning. Learn. Mem. 9, 138–150. 10.1101/lm.46502 12075002 PMC182592

[B4] Baess P. Horvath J. Jacobsen T. Schröger E. (2011). Selective suppression of self-initiated sounds in an auditory stream: an ERP study. Psychophysiology 48, 1276–1283. 10.1111/j.1469-8986.2011.01196.x 21449953

[B5] Balleine B. W. Ostlund S. B. (2007). Still at the choice-point: action selection and initiation in instrumental conditioning. Ann. N Y Acad. Sci. 1104, 147–171. 10.1196/annals.1390.006 17360797

[B6] Blakemore S. J. Wolpert D. M. Frith C. D. (1998). Central cancellation of self-produced tickle sensation. Nat. Neurosci. 1, 635–640. 10.1038/2870 10196573

[B7] Brem S. Lang-Dullenkopf A. Maurer U. Halder P. Bucher K. Brandeis D. (2005). Neurophysiological signs of rapidly emerging visual expertise for symbol strings. Neuroreport 16, 45–48. 10.1097/00001756-200501190-00011 15618888

[B8] Clark K. Appelbaum L. G. van den Berg B. Mitroff S. R. Woldorff M. G. (2015). Improvement in visual search with practice: mapping learning-related changes in neurocognitive stages of processing. J. Neurosci. 35, 5351–5359. 10.1523/JNEUROSCI.1152-14.2015 25834059 PMC4381005

[B9] Colwill R. M. Rescorla R. A. (1985). Postconditioning devaluation of a reinforcer affects instrumental responding. J. Exp. Psychol. Anim. Behav. Process. 11, 120–132. 10.1037/0097-7403.11.1.120

[B11] Desantis A. Hughes G. Waszak F. (2012). Intentional binding is driven by the mere presence of an action and not by motor prediction. PLoS One 7:e29557. 10.1371/journal.pone.0029557 22272237 PMC3260140

[B10] de Wit S. Dickinson A. (2009). Associative theories of goal-directed behaviour: a case for animal-human translational models. Psychol. Res. 73, 463–476. 10.1007/s00426-009-0230-6 19350272 PMC2694930

[B12] Dickinson A. Balleine B. (1994). Motivational control of goal-directed action. Anim. Learn. Behav. 22, 1–18. 10.3758/bf03199951

[B13] Elsner B. Hommel B. (2001). Effect anticipation and action control. J. Exp. Psychol. Hum. Percept. Perform. 27, 229–240. 10.1037/0096-1523.27.1.229 11248937

[B14] Estévez A. F. Fuentes L. J. Mari-Beffa P. González C. Alvarez D. (2001). The differential outcome effect as a useful tool to improve conditional discrimination learning in children. Learn. Motiv. 32, 48–64. 10.1006/lmot.2000.1060

[B15] Fort A. Delpuech C. Pernier J. Giard M. H. (2002). Early auditory-visual interactions in human cortex during nonredundant target identification. Cogn. Brain Res. 14, 20–30. 10.1016/s0926-6410(02)00058-7 12063127

[B16] Greenwald A. G. (1970a). A choice reaction time test of ideomotor theory. J. Exp. Psychol. 86, 20–25. 10.1037/h0029960 5482033

[B17] Greenwald A. G. (1970b). Sensory feedback mechanisms in performance control—with special reference to ideo-motor mechanism. Psychol. Rev. 77, 73–99. 10.1037/h0028689 5454129

[B18] Haggard P. Clark S. Kalogeras J. (2002). Voluntary action and conscious awareness. Nat. Neurosci. 5, 382–385. 10.1038/nn827 11896397

[B19] Herwig A. Prinz W. Waszak F. (2007). Two modes of sensorimotor integration in intention-based and stimulus-based actions. Q. J. Exp. Psychol. (Hove) 60, 1540–1554. 10.1080/17470210601119134 17853217

[B20] Hommel B. (1996). The cognitive representation of action: automatic integration of perceived action effects. Psychol. Res. 59, 176–186. 10.1007/bf00425832 8923816

[B21] Houde J. F. Nagarajan S. S. Sekihara K. Merzenich M. M. (2002). Modulation of the auditory cortex during speech: an MEG study. J. Cogn. Neurosci. 14, 1125–1138. 10.1162/089892902760807140 12495520

[B22] Hughes G. Desantis A. Waszak F. (2013a). Attenuation of auditory N1 results from identity-specific action-effect prediction. Eur. J. Neurosci. 37, 1152–1158. 10.1111/ejn.12120 23331545

[B23] Hughes G. Desantis A. Waszak F. (2013b). Mechanisms of intentional binding and sensory attenuation: the role of temporal prediction, temporal control, identity prediction, and motor prediction. Psychol. Bull. 139, 133–151. 10.1037/a0028566 22612280

[B24] Klem G. H. Lüders H. O. Jasper H. H. Elger C. (1999). The ten-twenty electrode system of the international federation. the international federation of clinical neurophysiology. Electroencephalogr. Clin. Neurophysiol. Suppl. 52, 3–6. 10590970

[B25] Krieghoff V. Waszak F. Prinz W. Brass M. (2011). Neural and behavioral correlates of intentional actions. Neuropsychologia 49, 767–776. 10.1016/j.neuropsychologia.2011.01.025 21255591

[B26] Kühn S. Seurinck R. Fias W. Waszak F. (2010). The internal anticipation of sensory action effects: when action induces FFA and PPA activity. Front. Hum. Neurosci. 4:12. 10.3389/fnhum.2010.00054 20661462 PMC2907885

[B27] Kunde W. (2001). Response-effect compatibility in manual choice reaction tasks. J. Exp. Psychol. Hum. Percept. Perform. 27, 387–394. 10.1037/0096-1523.27.2.387 11318054

[B28] Lange K. (2011). The reduced N1 to self-generated tones: an effect of temporal predictability? Psychophysiology 48, 1088–1095. 10.1111/j.1469-8986.2010.01174.x 21261634

[B29] Le Pelley M. E. McLaren I. P. (2003). Learned associability and associative change in human causal learning. Q. J. Exp. Psychol. B 56, 68–79. 10.1080/02724990244000179 12623538

[B30] Luck S. J. (2005). An Introduction to the Event-Related Potential Technique. Cambridge, MA: The MIT Press.

[B31] Mackintosh N. J. (1975). A theory of attention: variations in the associability of stimuli with reinforcement. Psychol. Rev. 82, 276–298. 10.1037/h0076778

[B32] Martikainen M. H. Kaneko K. Hari R. (2005). Suppressed responses to self-triggered sounds in the human auditory cortex. Cereb. Cortex 15, 299–302. 10.1093/cercor/bhh131 15238430

[B33] Martínez L. Estévez A. F. Fuentes L. J. Overmier J. B. (2009). Improving conditional discrimination learning and memory in five-year-old children: differential outcomes effect using different types of reinforcement. Q. J. Exp. Psychol. 62, 1617–1630. 10.1080/17470210802557827 19214832

[B34] McCarthy G. Donchin E. (1976). The effects of temporal and event uncertainty in determining the waveforms of the auditory event related potential (ERP). Psychophysiology 13, 581–590. 996223 10.1111/j.1469-8986.1976.tb00885.x

[B35] Melcher T. Winter D. Hommel B. Pfister R. Dechent P. Gruber O. (2013). The neural substrate of the ideomotor principle revisited: evidence for asymmetries in action-effect learning. Neuroscience 231, 13–27. 10.1016/j.neuroscience.2012.11.035 23206874

[B36] Mifsud N. G. Oestreich L. K. Jack B. N. Ford J. M. Roach B. J. Mathalon D. H.. (2016). Self-initiated actions result in suppressed auditory but amplified visual evoked components in healthy participants. Psychophysiology 53, 723–732. 10.1111/psyp.12605 26751981

[B37] Mishra J. Rolle C. Gazzaley A. (2015). Neural plasticity underlying visual perceptual learning in aging. Brain Res. 1612, 140–151. 10.1016/j.brainres.2014.09.009 25218557 PMC4362864

[B38] Mohr H. Wolfensteller U. Frimmel S. Ruge H. (2015). Sparse regularization techniques provide novel insights into outcome integration processes. Neuroimage 104, 163–176. 10.1016/j.neuroimage.2014.10.025 25467302

[B39] Mok L. W. Overmier J. B. (2007). The differential outcomes effect in normal human adults using a concurrent-task within-subjects design and sensory outcomes. Psychol. Rec. 57, 187–200.

[B40] Molholm S. Ritter W. Javitt D. C. Foxe J. J. (2004). Multisensory visual-auditory object recognition in humans: a high-density electrical mapping study. Cereb. Cortex 14, 452–465. 10.1093/cercor/bhh007 15028649

[B41] Murray M. M. Thelen A. Thut G. Romei V. Martuzzi R. Matusz P. J. (2016). The multisensory function of the human primary visual cortex. Neuropsychologia 83, 161–169. 10.1016/j.neuropsychologia.2015.08.011 26275965

[B42] Noonan M. P. Mars R. B. Rushworth M. F. S. (2011). Distinct roles of three frontal cortical areas in reward-guided behavior. J. Neurosci. 31, 14399–14412. 10.1523/JNEUROSCI.6456-10.2011 21976525 PMC3224993

[B43] Oostenveld R. Fries P. Maris E. Schoffelen J. M. (2011). FieldTrip: open source software for advanced analysis of MEG, EEG, and invasive electrophysiological data. Comput. Intell. Neurosci. 2011:156869. 10.1155/2011/156869 21253357 PMC3021840

[B44] Pfister R. Kiesel A. Hoffmann J. (2011). Learning at any rate: action-effect learning for stimulus-based actions. Psychol. Res. 75, 61–65. 10.1007/s00426-010-0288-1 20490862

[B45] Pfister R. Kiesel A. Melcher T. (2010). Adaptive control of ideomotor effect anticipations. Acta Psychol. 135, 316–322. 10.1016/j.actpsy.2010.08.006 20875631

[B46] Pfister R. Melcher T. Kiesel A. Dechent P. Gruber O. (2014). Neural correlates of ideomotor effect anticipations. Neuroscience 259, 164–171. 10.1016/j.neuroscience.2013.11.061 24333210

[B47] Reznik D. Henkin Y. Schadel N. Mukamel R. (2014). Lateralized enhancement of auditory cortex activity and increased sensitivity to self-generated sounds. Nat. Commun. 5:4059. 10.1038/ncomms5059 24898564

[B50] Ruge H. Krebs R. M. Wolfensteller U. (2012). Early markers of ongoing action-effect learning. Front. Psychol. 3:522. 10.3389/fpsyg.2012.00522 23205016 PMC3506999

[B51] Ruge H. Müller S. C. Braver T. S. (2010). Anticipating the consequences of action: an fMRI study of intention-based task preparation. Psychophysiology 47, 1019–1027. 10.1111/j.1469-8986.2010.01027.x 20477978 PMC3268076

[B48] Ruge H. Wolfensteller U. (2013). Functional integration processes underlying the instruction-based learning of novel goal-directed behaviors. Neuroimage 68, 162–172. 10.1016/j.neuroimage.2012.12.003 23246992

[B49] Ruge H. Wolfensteller U. (2015). Distinct fronto-striatal couplings reveal the double-faced nature of response-outcome relations in instruction-based learning. Cogn. Affect. Behav. Neurosci. 15, 349–364. 10.3758/s13415-014-0325-4 25361755 PMC4436102

[B52] SanMiguel I. Todd J. Schröger E. (2013). Sensory suppression effects to self-initiated sounds reflect the attenuation of the unspecific N1 component of the auditory ERP. Psychophysiology 50, 334–343. 10.1111/psyp.12024 23351131

[B53] Schafer E. W. Marcus M. M. (1973). Self-stimulation alters human sensory brain responses. Science 181, 175–177. 10.1126/science.181.4095.175 4711735

[B54] Shin Y. K. Proctor R. W. (2012). Testing boundary conditions of the ideomotor hypothesis using a delayed response task. Acta Psychol. 141, 360–372. 10.1016/j.actpsy.2012.09.008 23089044

[B55] Song Y. Ding Y. Fan S. Qu Z. Xu L. Lu C.. (2005). Neural substrates of visual perceptual learning of simple and complex stimuli. Clin. Neurophysiol. 116, 632–639. 10.1016/j.clinph.2004.09.019 15721077

[B56] Talsma D. Doty T. J. Woldorff M. G. (2007). Selective attention and audiovisual integration: is attending to both modalities a prerequisite for early integration? Cereb. Cortex 17, 679–690. 10.1093/cercor/bhk016 16707740

[B57] Timm J. SanMiguel I. Keil J. Schröger E. Schönwiesner M. (2014). Motor intention determines sensory attenuation of brain responses to self-initiated sounds. J. Cogn. Neurosci. 26, 1481–1489. 10.1162/jocn_a_00552 24392902

[B58] Timm J. Schönwiesner M. Schröger E. SanMiguel I. (2016). Sensory suppression of brain responses to self-generated sounds is observed with and without the perception of agency. Cortex 80, 5–20. 10.1016/j.cortex.2016.03.018 27137101

[B59] Trapold M. A. (1970). Are expectancies based upon different positive reinforcing events discriminably different. Learn. Motiv. 1, 129–140. 10.1016/0023-9690(70)90079-2

[B60] Trapold M. A. Overmier J. B. (1972). “The second learning process in instrumental learning,” in Classical Conditioning II: Current Research and Theory, eds Black A. H. Prokasy W. F. (New York, NY: Appleton-Cwntury-Croft), 427–452.

[B61] Urcuioli P. J. (2005). Behavioral and associative effects of differential outcomes in discrimination learning. Learn. Behav. 33, 1–21. 10.3758/bf03196047 15971490

[B62] Von Holst E. Mittelstaedt H. (1950). Das reafferenzprinzip—(Wechselwirkungen zwischen zentralnervensystem und peripherie). Naturwissenschaften 37, 464–476. 10.1007/BF00622503

[B63] Waszak F. Cardoso-Leite P. Hughes G. (2012). Action effect anticipation: neurophysiological basis and functional consequences. Neurosci. Biobehav. Rev. 36, 943–959. 10.1016/j.neubiorev.2011.11.004 22108008

[B64] Waszak F. Wascher E. Keller P. Koch I. Aschersleben G. Rosenbaum D. A.. (2005). Intention-based and stimulus-based mechanisms in action selection. Exp. Brain Res. 162, 346–356. 10.1007/s00221-004-2183-8 15599722

[B65] Weiskrantz L. Elliott J. Darlington C. (1971). Preliminary observations on tickling oneself. Nature 230, 598–599. 10.1038/230598a0 4928671

[B66] Wolfensteller U. Ruge H. (2011). On the timescale of stimulus-based action-effect learning. Q. J. Exp. Psychol. 64, 1273–1289. 10.1080/17470218.2010.546417 21416458

[B67] Wolfensteller U. Ruge H. (2012). Frontostriatal mechanisms in instruction-based learning as a hallmark of flexible goal-directed behavior. Front. Psychol. 3:192. 10.3389/fpsyg.2012.00192 22701445 PMC3371695

[B68] Wolfensteller U. Ruge H. (2014). Response selection difficulty modulates the behavioral impact of rapidly learnt action effects. Front. Psychol. 5:1382. 10.3389/fpsyg.2014.01382 25566108 PMC4266035

[B69] Zwosta K. Ruge H. Wolfensteller U. (2015). Neural mechanisms of goal-directed behavior: outcome-based response selection is associated with increased functional coupling of the angular gyrus. Front. Hum. Neurosci. 9:180. 10.3389/fnhum.2015.00180 25914635 PMC4392699

